# Orbit Determination of Korean GEO Satellite Using Single SLR Sensor

**DOI:** 10.3390/s18092847

**Published:** 2018-08-29

**Authors:** Hyungjik Oh, Eunseo Park, Hyung-Chul Lim, Chandeok Park

**Affiliations:** 1 Department of Astronomy, Yonsei University, Seoul 03722, Korea; jayoh@yonsei.ac.kr; 2 Korea Astronomy and Space Science Institute, Daejeon 34055, Korea; skel93@kasi.re.kr (E.P.); hclim@kasi.re.kr (H.-C.L.); 3 Department of Astronomy & Yonsei University Observatory, Yonsei University, Seoul 03722, Korea

**Keywords:** GK-2B, Compass-G1, geostationary Earth orbit (GEO), orbit determination (OD), satellite laser ranging (SLR)

## Abstract

Geostationary Earth Orbit (GEO)-Korea Multi-Purpose Satellite (KOMPSAT)-2B (GK-2B) is a Korean geostationary Earth orbit (GEO) satellite that is scheduled to be launched in 2020 for meteorological and ocean monitoring. While the primary orbit determination (OD) for GK-2B is by ground-based radar observations and the expected orbit precision is less than 1 km, a satellite laser ranging (SLR) technique has been selected as a subsidiary OD method to verify/complement/enhance primary OD results. In general, the available time and equipment for observing GEO satellites with SLR are limited. Furthermore, because the optical sensors mounted on GK-2B may be defected by laser, only a domestic single SLR station would obtain the tracking data. This research presents the mitigation of these drawbacks to improve orbit precision. Observation data generation and the associated OD of GK-2B are performed by considering numerical SLR data analysis on Compass-G1, a Chinese GEO navigation satellite, and Chinese SLR station at Changchun. With the OD performed for two scenarios with the varying number of observations, the 3D position error is 24.01 m when 13 observations per day are obtained, while the error becomes 43.46 m when 9 observations per day are obtained. To verify these results, the OD of Compass-G1 using actual SLR data from Changchun station is performed to yield 31.89 m for 3D error, which is favorable compared with the external precise ephemeris by GeoForschungsZentrum (GFZ) analysis center. Therefore, the OD based on single SLR station is applicable to estimating the orbit within less than 100 m.

## 1. Introduction

Geostationary Earth Orbit (GEO)-Korea Multi-Purpose Satellite (KOMPSAT) 2 (GK-2) is a space program that is a part of the Korea Multi-Purpose Satellite (KOMPSAT) project by Korea Aerospace Research Institute (KARI) for meteorological and ocean monitoring by two geostationary Earth orbit (GEO) satellites. It is a follow-up mission to the GEO-KOMPSAT-1 (GK-1), which was launched in 2010 and has successfully provided high-resolution aerosol information above east Asia with the meteorological imager (MI) and the geostationary ocean color imager (GOCI) [1]. In the GK-2 program, two satellites, GK-2A and GK-2B, will be ready to be launched in 2018 and 2020, respectively. GK-2A aims at meteorological observation and space weather monitoring with an advanced meteorological imager (AMI) and the Korean Space Environment Monitor (KSEM). Meanwhile, the GOCI-II and geostationary environmental monitoring sensor (GEMS) will be mounted on GK-2B for ocean and environmental monitoring [2].

As the number of GEO satellites increases, orbit determination (OD) and monitoring of GEO satellites become essential to avoid collision between satellites [3,4]. For GK-2B, ground-based radar is selected as a primary observation for OD, and the requirement of orbit precision is 1 km. Hwang et al. [5] performed the OD of GK-1 with single station antenna to yield position accuracy of 1.5 km. Choi et al. [6] conducted OD by angles-only optical tracking data of GK-1 for 2-day arc to have less than 2 km maximum difference from two-line elements (TLEs). Unlike GK-2A, GK-2B will be equipped with a laser retro-reflector array (LRA) to acquire satellite laser ranging (SLR) observation data. As one of the most precise ranging techniques [7], SLR has been widely applied to the satellite OD. However, most of the previous studies about SLR-based OD focused on satellites located on low Earth orbit (LEO) and medium Earth orbit (MEO). For GEO satellites, SLR is typically applied to assess the quality of OD solution by calculating the range residual between estimated orbit and SLR data due to the limitations of SLR observations. Furthermore, only a few SLR stations that possess a high power laser transmitter can obtain SLR observations for objects at high altitude, such as GEO satellites. Because of the little transition in the ground trajectory of GEO satellites, the location of the SLR stations that can observe a specific satellite is limited. In addition to the aforementioned problems, SLR stations capable of observing GEO satellites can obtain the SLR observations only at night. These challenging situations make it difficult to acquire SLR observations of GEO satellites in contrast to the case of LEO/MEO satellites, resulting in deficiency in observation data for satellite OD. Nevertheless, by considering that the SLR observations are relatively accurate compared with other observations, SLR-based OD is still applicable to verify estimated orbit by other observations and to improve the orbit precision.

Previous studies intermittently determined the orbit of GEO satellites using SLR measurements. Oh et al. [8] conducted OD of high Earth orbit (HEO) satellites using SLR measurements. For the selected Japanese Quasi-Zenith Satellite-1 (QZS-1) and Chinese Compass-G1, the post-fit root-mean-square (RMS) residuals of OD were tens of centimeters. Zhao et al. [9] obtained orbits of BeiDou satellites with meter-level accuracy using SLR observation data. Unlike the OD of other GEO satellites, the OD of GK-2B satellite needs to impose additional constraints that were not considered in previous studies. First, in order for satellite-mounted optical instruments not to be damaged by laser during the observation, only one domestic SLR station is employed to obtain SLR observation data for GK-2B. This restriction has been previously imposed for several satellite missions such as advanced land observing satellite (ALOS); the ice, cloud, and land elevation satellite (ICESat); Lomonosov; and Sentinel-3A. As Korea only has one available SLR station to obtain observation data of GEO satellites in Geochang, the OD of GEO satellites based on a single SLR station must be conducted.

**Figure 1:**
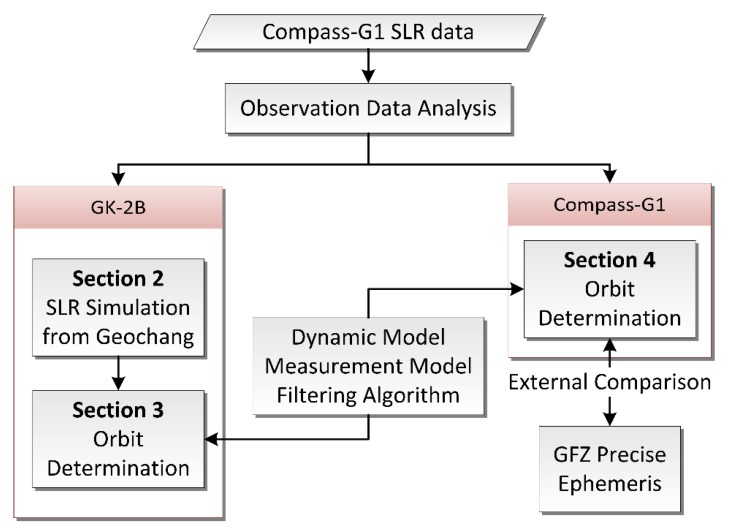
Orbit determination procedure. SLR—satellite laser ranging; GFZ—GeoForschungsZentrum.

This study presents the OD of GK-2B based on a single station that satisfies the accuracy requirement. Figure 1 summarizes the flowchart of the OD procedure. The paper is organized as follows: In Section 2, SLR observation data simulation for GK-2B from Geochang SLR station is performed. In an effort to mimic the actual GEO satellite observations as closely as possible in the simulation, the observation data is generated based on quantitative observation data analysis for Compass-G1 GEO satellite. Two scenarios are configured with different numbers of SLR observations. In Section 3, OD of GK-2B based on simulated SLR observations is conducted for two scenarios. Also, to verify the validity of the derived OD strategy, the OD for Compass-G1 based on actual SLR observations from Changchun station is performed in Section 4. As Compass-G1 is part of the Global Navigation Satellite System (GNSS), a final precise orbit generated from International GNSS service (IGS) Analysis Center (AC) is obtainable. The estimated orbit from the OD process is externally compared with the final orbit from one of IGS ACs and the resultant orbit overlap errors are quantitatively analyzed. Section 5 draws conclusions.

## 2. SLR Data Generation

**Figure 2:**
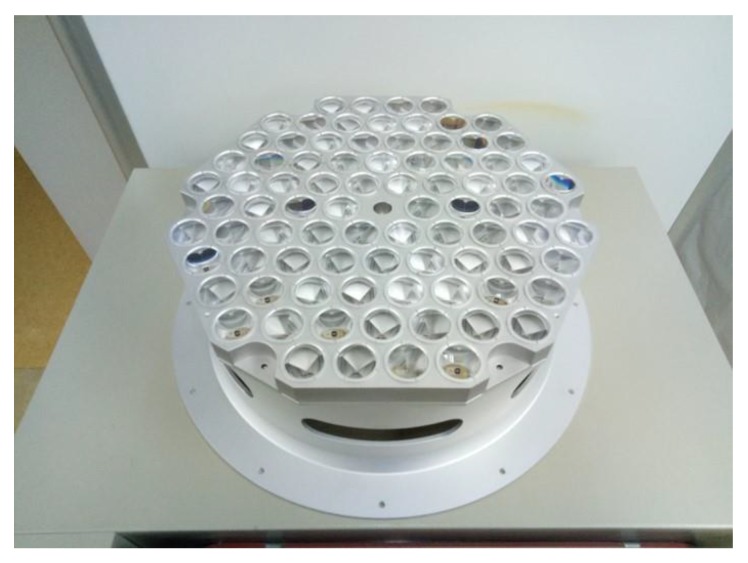
Laser retro-reflector array of Geostationary Earth Orbit (GEO)-Korea Multi-Purpose Satellite (KOMPSAT)-2B (GK-2B).

The orbit of GK-2B has been fixed to GEO of 128.408° east longitude to satisfy its primary purpose of monitoring meteorological and marine environments around the Korean peninsula. Figure 2 shows the LRA flight model of GK-2B. The diameter of each of 84 corner cube retro-reflector (CCR) is 33 mm. Total reflective area for the LRA is set to 718 cm^2^ and the weight is about 9.54 kg, which is relatively large compared with that of common LRA for LEO/MEO satellites because of the long distance to ground stations.

**Figure 3:**
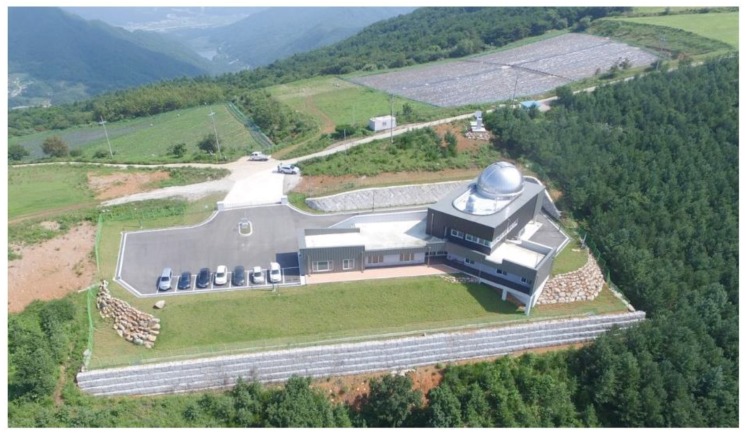
Geochang SLR station on Mt. Gamak.

**Figure 4:**
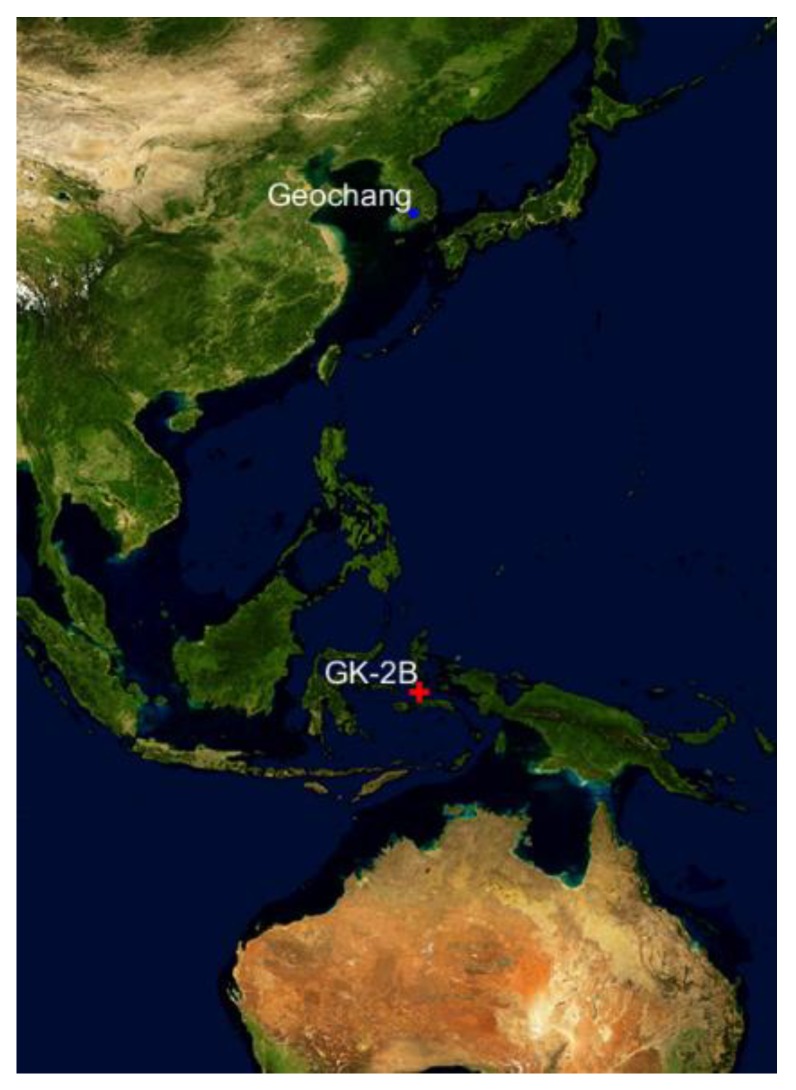
Ground trajectory of GK-2B (red cross) and Geochang station (blue dot).

Geochang station was constructed at Gamak Mountain by Korea Astronomy and Space Science Institute (KASI) as the second Korean SLR system. It is in pilot operation and calibration stage and is expected to operate normally in 2019. Figure 3 shows the overview of Geochang station. Unlike the first Korean SLR system located in Sejong, the laser intensity of Geochang system is high enough to obtain SLR observations for high-altitude satellites. The system also aims to perform debris laser tracking (DLT) along with SLR, and the beam diameter of transmitting telescope for the laser system is 100 cm, which is ten times larger than that of Sejong station. Also, adaptive optics (AO) is applied to the Geochang system to obtain satellite images larger than 10 magnitudes [10]. Figure 4 shows the ground trajectory of GK-2B and the location of Geochang SLR station.

Table 1 presents the detailed mission parameters of GK-2B. The mass of the satellite decreases from 1925 kg at the beginning of life to 1492 kg at the end of life because of fuel consumption. The satellite position and velocity state vectors in this paper are set on 28 June 2020 with true-of-date (TOD) coordinate system. Table 2 shows the station coordinates of Geochang in the geodetic coordinate system. For simulating SLR observations, Orbit Determination Toolkit (ODTK) version 6 of Analytical Graphics Inc. (AGI, Washington, DC, USA), is utilized. Once the orbit of the satellite and SLR ground station is provided, the program generates simulated range-only SLR observations by adding Gaussian random noise into the true range between the target satellite and the SLR station. The one sigma error of SLR observations is set to 4 mm.

**Figure 5:**
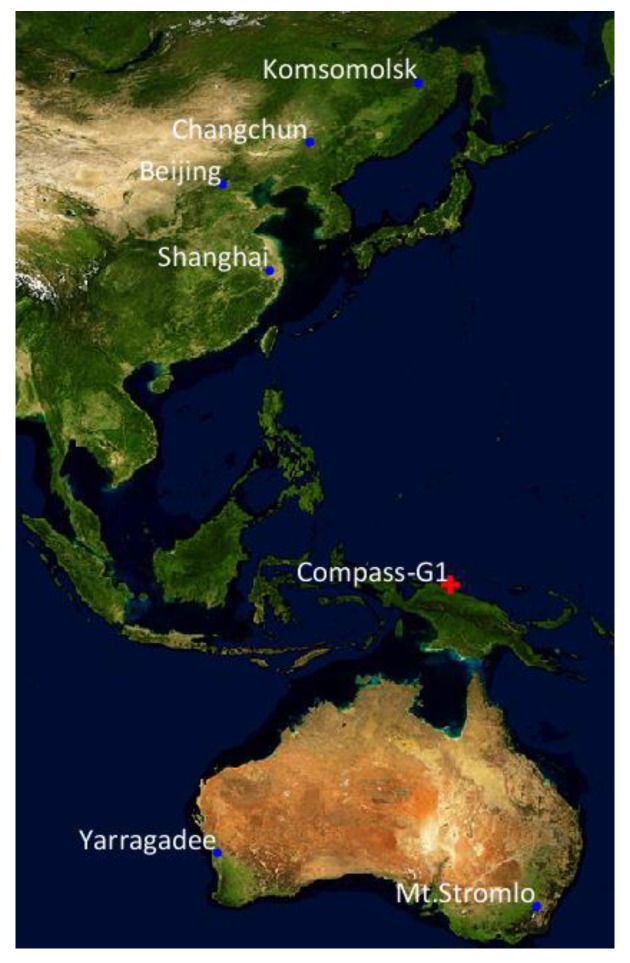
Ground trajectory of Compass-G1 (red cross) and SLR stations (blue dots).

As mentioned in the Introduction, obtaining SLR observations for GEO satellites is limited. For the observation data simulation, considering all these factors and figuring out how many actual SLR observations of GK-2B will be available in the future, statistical analysis is performed on the SLR observations of a GEO satellite in operation. The selected operational GEO satellite is Compass-G1 of BeiDou Navigation System (BDS). Figure 5 shows the ground trajectory of the satellite and the location of SLR stations that were successful in observing Compass-G1 in 2017. Six stations, including three Chinese stations, obtained SLR data of Compass-G1 in this period.

**Figure 6:**
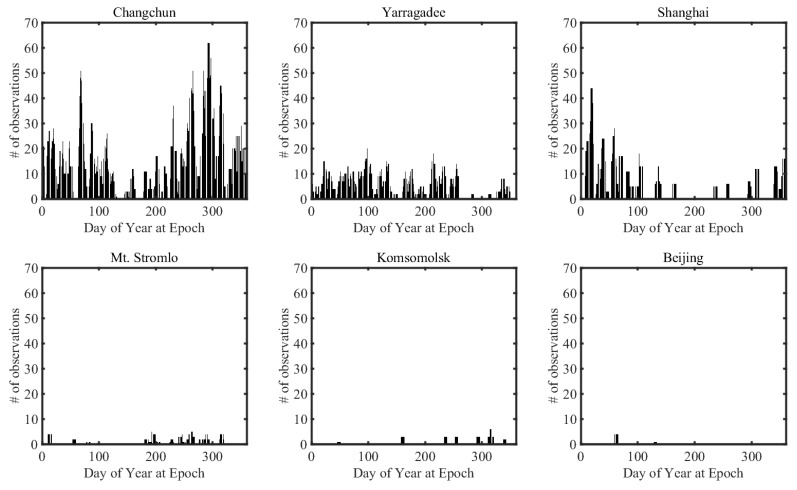
The number of Compass-G1 measurements in 2017.

Table 3 provides the number of observations of Compass-G1 by sorting the number of observations of each SLR stations into descending order. The type of SLR observations used in this study was normal point data. The number of passes indicates the number of attempts to track Compass-G1. Among those observations, Changchun station made the most SLR observations during the period, whereas Beijing station only obtained 10 SLR observations in the same period. The total observations in this period were 813 for Changchun, and the average number of measurements per one-day arc of about 2.227 was obtained by dividing by 365 days. Figure 6 shows the number of observations in one week-length arc with respect to the day of the year at the epoch. The arc that performed the most frequent observations of Compass-G1 in the whole period was from 21 October to 27 October at Changchun station. The number of observations was 62 in this period.

**Figure 7:**
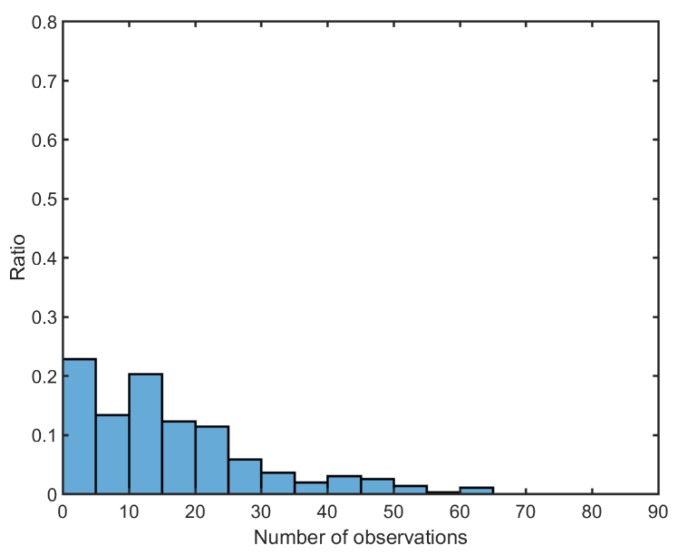
The ratio of the number of Compass-G1 observations at Changchun in 2017.

For more detailed analysis, the distribution of the number of observations in one arc is calculated as in Figure 7 and Table 4. The amount of observation data for each arc existing in 2017 is counted, and the results are divided into five observation intervals to calculate the distribution. The ratio of the interval of zero to four observations is the highest. Although many arcs are concentrated in the area where the number of observations is 25 or less, several arcs have more than 40 observations in an arc. In the arc of 62 observations, the observations were concentrated on the front part of the arc. There were 18, 10, 10, and 24 observations of Compass-G1 in Changchun station for 21, 22, 23, and 24 October, respectively. Therefore, it can be concluded from the analyses that more than 60 SLR observations for GK-2B will be available in one SLR station if the observation procedure is operated with no restricted conditions.

**Figure 8:**
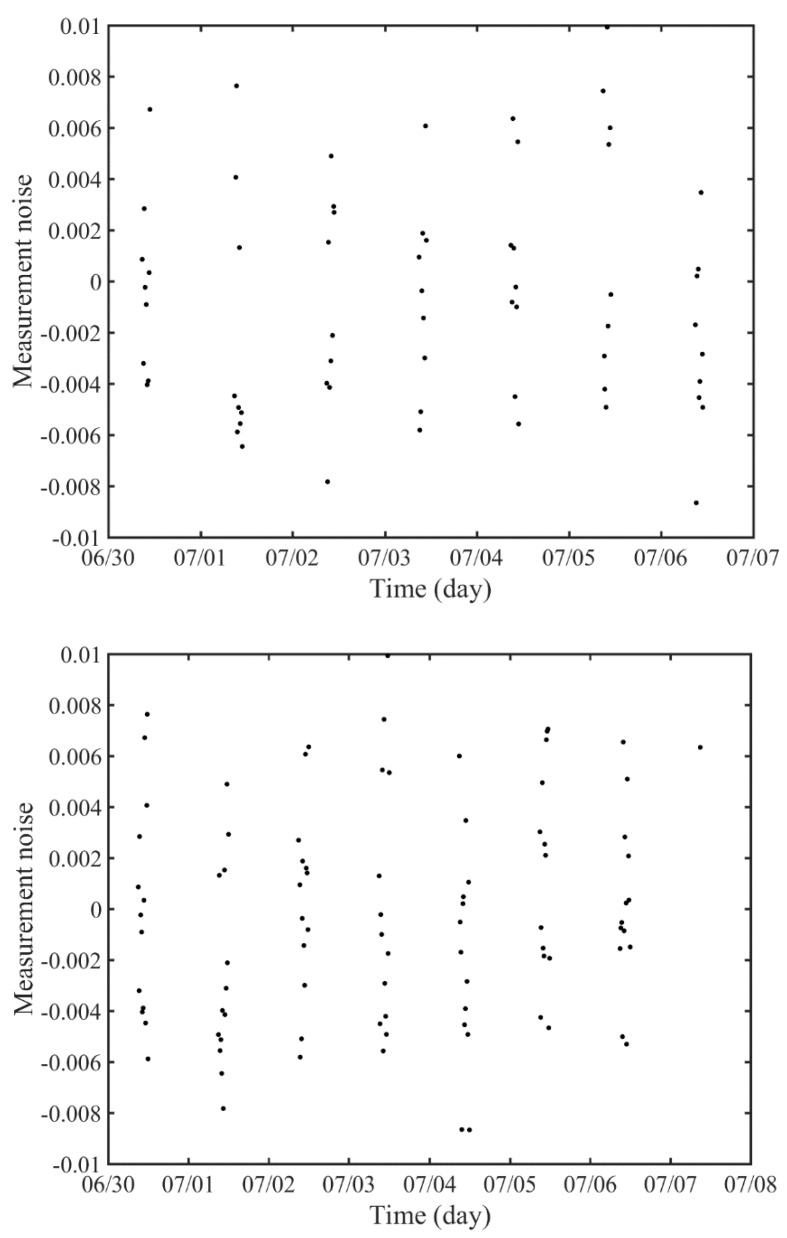
Simulated SLR observations for GK-2B from Geochang (**upper**: Scenario 1, **lower**: Scenario 2).

Based on statistical analyses of Compass-G1, Table 5 shows two observation scenarios. Scenario 1 set the generation of appropriate SLR observations for GK-2B to nine per one day, which results in 63 observations per week-length arc. The observation time is 2 h, spanning from midnight to 02:00, considering that GEO satellites can be only observed at night, and observation gap is 15 min. Scenario 2 extends the observation time into 3 h with the same observation gap, resulting in 13 observations per day and 98 observations per arc. The observation time for Scenario 2 spans from midnight to 03:00. Figure 8 represents simulated SLR observations for these two scenarios, in which the root-mean-square (RMS) of measurement noise is 4.316 mm. Unobservable periods are 22 h and 21 h for Scenario 1 and Scenario 2, respectively.

## 3. GK-2B Orbit Determination Using SLR Data

### 3.1. Orbit Determination Strategy

OD as well as SLR observation data generation is performed by ODTK, which utilizes a forward-running optimal sequential filter (like a Kalman filter) followed by the backward-running fixed-interval smoother process [12]. Details of mathematical theory for optimal filter and smoother are found in the literature [13]. Table 6 summarizes dynamic/observation model and its associated estimation parameters. The Global Gravity Model (GGM)03C model with degree and order of 30 is utilized for the Earth’s gravity. International Earth Rotation and Reference Systems Service (IERS) 1996 conventions accommodate the solid Earth tides, ocean tides, and general relativity correction. The Sun, Moon, and all planets in the Solar system are included for the third body gravity, and the orbital ephemeris comes from DE405 of Jet Propulsion Laboratory (JPL). The air drag model is not considered in the dynamic model because of the high altitude of GEO. The cross-sectional area for solar radiation pressure is set by spherical model, along with the eclipse occurring with the Earth and the Moon. The solar radiation coefficient is estimated in the filter by Gauss–Markov process. Empirical forces are set as one-cycle-per-rev model in three axes.

### 3.2. Orbit Determination

**Figure 9:**
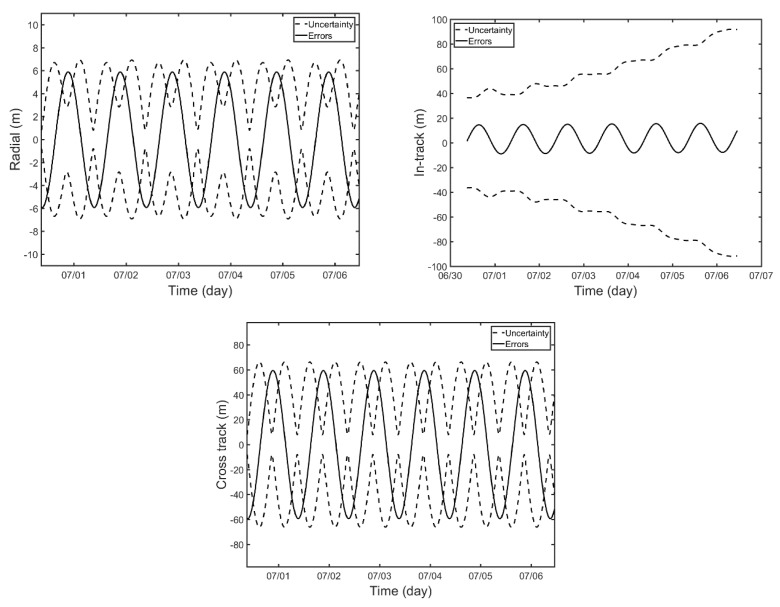
Estimated orbit uncertainty and orbital errors for Scenario 1 in radial (**upper left**), in-track (**upper right**), and cross-track (**lower**) directions.

OD of GK-2B is performed by iterating the forward sequential filter and backward smoothing process. Initial orbits are perturbed to 1 km in each direction for convergence in orbit. The solar radiation parameter included in the dynamic model is estimated, along with the six orbit states. Figure 9 shows the resultant position uncertainties of estimated orbit and orbit errors for Scenario 1. The diagonal term of the orbital covariance matrix indicates the orbit uncertainty. The estimated orbits are directly compared with the pre-generated true orbits in the simulation and represented in radial, in-track, and cross-track (RIC) frame. As a result of the characteristics of SLR observations, the orbital errors in radial direction are the smallest with respect to those of in-track and cross-track directions. The RMS errors in radial, in-track, and cross-track direction are 4.21 m, 8.94 m, and 42.32 m, respectively, and 43.46 m in three-dimension. While the errors in each direction show oscillation within some error bounds, the orbit uncertainty of in-track direction gradually increases over time while the position errors remain within ±20 m. 

**Figure 10:**
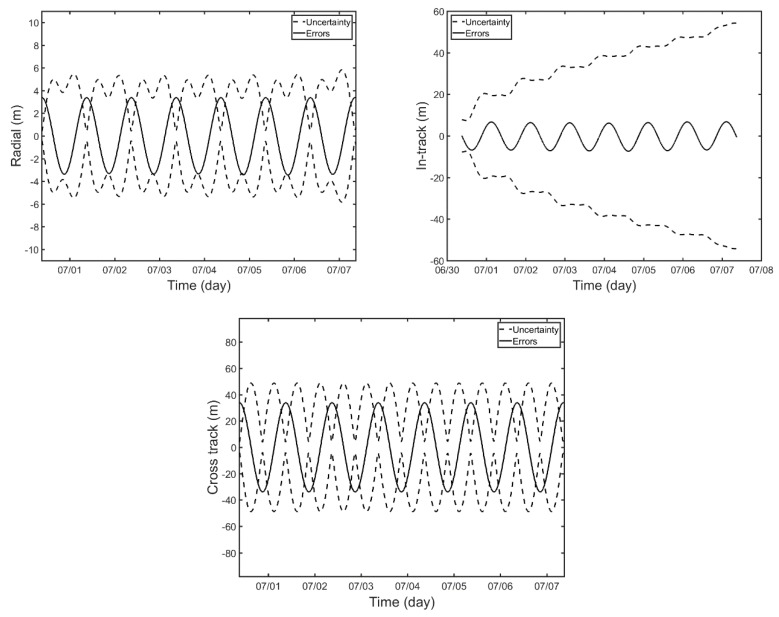
Estimated orbit uncertainty and orbital errors for Scenario 2 in radial (**upper left**), in-track (**upper right**), and cross-track (**lower**) directions.

Figure 10 shows the estimated orbit errors and uncertainty for Scenario 2. The errors in all directions show a slight decrease when compared with those of Scenario 1. The RMS errors are 2.40 m, 4.79 m, and 24.01 m for radial, in-track, cross-track direction, respectively. Three-dimensional orbit error is 24.60 m for the whole period. It is reduced from that of Scenario 1 by 56.6%. These results indicate that the OD precision could be enhanced with more frequent SLR observations.

## 4. Compass-G1 Orbit Determination for Strategy Verification

As the obtained OD solution of GK-2B is based on numerical simulation, the proposed OD remains to be validated for the OD in the real world. For instance, SLR data might include unexpected bias or errors caused by the local weather condition or unusual observation characteristics. Furthermore, the dynamic model utilized in the simulation does not accurately “simulate” the real-world dynamics. As an experimental verification of the proposed OD strategy, the OD based on actual observation is performed. The SLR observation data of Compass-G1 from the Changchun SLR station, which is the combination used in the observation analysis described in Section 2, are used as the verification target. As the arc with the most observation data in 2017 was from 21 October to 24 October, the OD is performed for that period. International Terrestrial Reference Frame (ITRF) 2014 coordinates are applied to the station coordinate system of Changchun. The other dynamic/observation model and estimation parameter settings are the same as those of GK-2B OD simulation.

**Figure 11:**
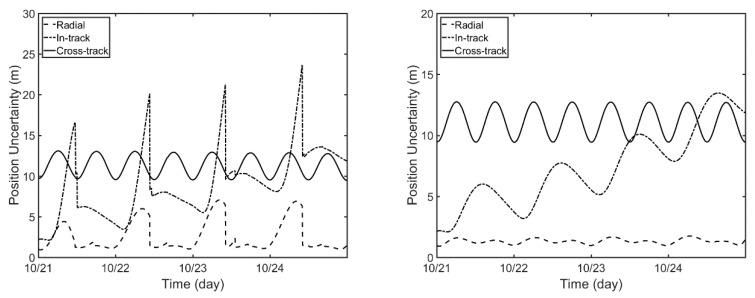
Position uncertainty of filter (**left**) and smoother (**right**) from Compass-G1 orbit determination (OD).

Figure 11 describes the position uncertainty from the filter/smoother in the Compass-G1 OD process. The position uncertainty is reduced when the sufficient observation data are obtained, while it continuously increases without observations for radial and in-track directions. After the smoothing process as shown on the right-hand side of Figure 11, the position uncertainty peaks mostly disappear for the whole arc. The RMS of position uncertainty in the filter is 3.18 m, 10.01 m, and 11.38 m, for radial, in-track, and cross-track directions, respectively, and these decrease to 1.33 m, 8.00 m, and 11.20 m, respectively, after the smoothing process.

**Figure 12:**
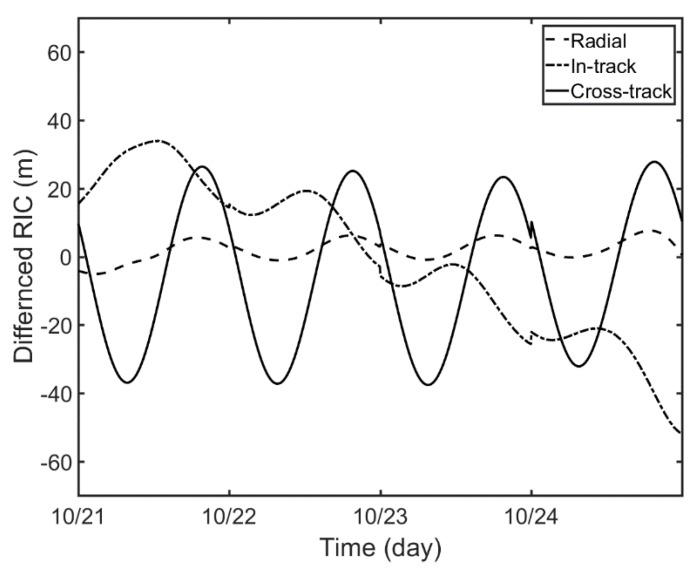
Orbit overlap differences between the estimated orbits and GeoForschungsZentrum (GFZ) final orbits.

The estimated orbits are now verified by external comparison. As Compass-G1 is a navigation satellite, the orbital ephemeris is regularly published online by IGS ACs. German GeoForschungsZentrum (GFZ) AC obtains precise ephemeris solution called the final orbit of Compass-G1. In general, the precision of the final orbit from GFZ is known to be 30–80 cm [18]. Figure 12 and Table 7 present the comparison between GFZ final orbit and the estimated orbit from this study in radial, in-track, and cross-track directions. The GFZ publishes the final orbit of Compass-G1 with 5 min intervals. Therefore, the orbit overlap comparison is performed every 5 min for the whole four-day arc. The errors in the radial direction are the smallest while the errors in in-track and cross-track directions are about 22 m. From these comparative analyses, it can be concluded that the OD of GEO satellites based on a single SLR station is applicable under normal observation environments. The orbit precision is enhanced to tens of meters, which is a vast improvement compared with the OD with other observations such as optical tracking or angles-only data.

## 5. Conclusions

In this study, orbit determination (OD) of geostationary Earth orbit (GEO) satellite was performed for strategy development of OD of Korean GK-2B satellite under the limited SLR observations from a single satellite laser ranging (SLR) station. As Geochang station is the only domestic SLR station that can obtain SLR data for GEO satellites, SLR observation data for GK-2B from this station have been generated. Then, the OD was conducted by sequential filtering followed by smoothing process repeatedly for a week-long arc to yield 43.46 m and 24.01 m in 3D RMS errors for the sparse and dense case, respectively. As a validation of the proposed OD strategy, actual SLR observations of Compass-G1 by Changchun SLR station were utilized. The associated orbit overlap errors compared with Compass-G1 final orbit from GFZ AC were 3.747 m, 22.375 m, and 22.406 m, in radial, in-track, and cross-track directions, respectively, and the 3D RMS error was 31.89 m. These OD results imply that the orbit accuracy can remain within 50 m when more than nine SLR observations are available for a single SLR station. Given that the radar-based OD achieved three-sigma position accuracy on the order of 1.5 km [5], and the optical-tracking-based OD precision for GEO satellite was about km-level [6], SLR can be an alternative observation for enhancing OD of GEO satellites.

In contrast to previous requirements on the OD of GEO, precise OD of GEO satellites becomes more important with the increasing number of Regional Navigation Satellite Systems (RNSSs), because the orbit requirement of navigation satellites is relatively tight compared with that of general GEO satellites for communication/ground monitoring purpose. Because GEO satellites are always visible in the specific ground region, many Regional Navigation Satellite System (RNSS), such as Chinese BeiDou Navigation System (BDS), Indian Regional Navigation Satellite System (IRNSS), and Japanese Quasi-Zenith Satellite System (QZSS), include GEO satellites in their orbit constellation. Korea also plans to construct its own domestic RNSS in the future, and three GEO satellites will be in constellation. SLR is expected to play a more critical role in OD of these GEO navigation satellites and the OD strategy developed in this article is applicable.

## Figures and Tables

**Table 1:** Geostationary Earth Orbit (GEO)-Korea Multi-Purpose Satellite (KOMPSAT)-2B (GK-2B) mission parameters [11]. UTC—Coordinated Universal Time.

Parameter	Value
Development period	July 2011–September 2019
Year of launch	2020
Mission lifetime	10 years
Primary mission	Meteorological observation Space weather monitoring
Satellite mass	1925 kg @BOL ^1^, 1492 kg @EOL ^2^
Satellite cross section	22.68 m^2^
Surface reflectance	0.41
Epoch orbit (UTC 09:00:00 29 June 2020)	***r*** = [−42,160.2970 −613.6082 6.9611] km ***v*** = [0.044454 −3.07440 −0.00107] km/s

^1^ Beginning of life; ^2^ end of life

**Table 2:** Geochang station information. SLR—satellite laser ranging; DLT—debris laser tracking.

Parameter	Value
Latitude	35.5901665597°
Longitude	127.920064752°
Height	934.063 m
Repetition rate	60 Hz nominal for SLR 10 Hz for DLT
One sigma error for range measurements	4 mm

**Table 3:** SLR tracking statistics of Compass-G1 in 2017.

Station Name	Station Number	No. Passes	No. Observations
Changchun	7237	270	813
Yarragadee	7090	106	288
Shanghai	7821	66	277
Mount Stromlo	7825	20	43
Komsomolsk	1868	9	26
Beijing	7249	3	10

**Table 4:** The ratio of the number of Compass-G1 measurements at Changchun in 2017.

# of Observations	Ratio (%)	# of Observations	Ratio (%)
0–4	22.84	35–39	1.95
5–9	13.37	40–44	3.06
10–14	20.33	45–49	2.51
15–19	12.26	50–54	1.39
20–24	11.42	55–59	0.28
25–29	5.85	60–64	1.11
30–34	3.62	65–69	0

**Table 5:** GK-2B orbit determination (OD) scenario.

Scenario	Observation Time	Observation Gap
1	2 h a day	15 min
2	3 h a day	15 min

**Table 6:** Dynamic models, observation models, and estimation parameters for GK-2B OD. IERS—International Earth Rotation and Reference Systems Service; JPL—Jet Propulsion Laboratory; GGM—Global Gravity Model.

Model/Parameter	Description
Earth gravity	GGM03C 30 × 30 [14]
Solid earth tides/Ocean tides/General relativity correction	IERS conventions 1996 [15]
Third body gravity	JPL DE405 ephemeris [16]
Air drag	None
Solar radiation pressure	Penumbral cone model [12]
Central body radiation	Earth albedo with thermal radiation pressure
Empirical forces	One-cycle-per-rev model [17]

**Table 7:** Orbit overlap differences of Compass-G1.

Direction	Radial (m)	In-Track (m)	Cross-Track (m)
Overlap errors	3.747	22.375	22.406

## Abbreviations

AC	Analysis Center
AO	Adaptive Optics
AGI	Analytical Graphics Inc.
ALOS	Advanced Land Observing Satellite
AMI	Advanced Meteorological Imager
BDS	BeiDou Navigation System
BOL	Beginning of Life
CCR	Corner Cube Retro-reflector
DLT	Debris Laser Tracking
EOL	End of Life
GEMS	Geostationary Environmental Monitoring Sensor
GEO	Geostationary Earth Orbit
GFZ	GeoForschungsZentrum
GGM	Global Gravity Model
GK-1	GEO-KOMPSAT-1
GK-2	GEO-KOMPSAT-2
GK-2A	GEO-KOMPSAT-2A
GK-2B	GEO-KOMPSAT-2B
GNSS	Global Navigation Satellite System
GOCI	Geostationary Ocean Color Imager
HEO	High Earth Orbit
ICESat	Ice, Cloud, and land Elevation Satellite
IERS	International Earth Rotation and Reference Systems Service
IGS	International GNSS Service
IRNSS	Indian Regional Navigation Satellite System
ITRF	International Terrestrial Reference Frame
JPL	Jet Propulsion Laboratory
KARI	Korea Aerospace Research Institute
KASI	Korea Astronomy and Space Science Institute
KOMPSAT	Korea Multi-Purpose Satellite
KSEM	Korean Space Environment Monitor
LEO	Low Earth Orbit
LRA	Laser Retro-reflector Array
MEO	Medium Earth Orbit
MI	Meteorological Imager
OD	Orbit Determination
ODTK	Orbit Determination Toolkit
QZS-1	Quasi-Zenith Satellite-1
QZSS	Quasi-Zenith Satellite System
RIC	Radial, In-track, Cross-track
RMS	Root-Mean-Square
RNSS	Regional Navigation Satellite System
SLR	Satellite Laser Ranging
TLE	Two-Line Elements
TOD	True-of-Date
UTC	Coordinated Universal Time
